# Clinical Evaluation of the Safety and Tolerability of Film-Forming Sprays in Patients With Psoriasis and Eczema

**DOI:** 10.7759/cureus.57020

**Published:** 2024-03-27

**Authors:** Kiran Godse, Gautam Dethe, Shankar Sawant, Aseem Sharma, Rickson Pereira, Sunil Ghate, Sneha Kuvi, Varsha Pawar, Reshma Parekar, Maneesha Khalse, Kamlesh Patel

**Affiliations:** 1 Dermatology, D Y Patil Hospital, Navi Mumbai, IND; 2 Dermatology, Apollo Hospitals, Navi Mumbai, IND; 3 Dermatology, Rejoice Clinic, Mumbai, IND; 4 Dermatology, Skin Saga Centre for Dermatology, Mumbai, IND; 5 Dermatology, Dr. Rickson's Dermatherapie Clinic, Mumbai, IND; 6 Dermatology, Holy Family Hospital, Mumbai, IND; 7 Dermatology, Dr. Ghate's Skin and Laser Centre, Mumbai, IND; 8 Dermatology, Dr. Paruchuri Raja Ram Memorial Skin, Hair and Laser Centre, Guntur, IND; 9 Medical Affairs Division, Lupin Limited, Mumbai, IND

**Keywords:** skin disease, eczema, topical, occlusion, psoriasis, film forming spray, dermatology

## Abstract

Background

Treating dermatological pathologies under occlusion therapy is a popular adjunct, especially in thickened, lichenified areas of psoriasis where sustained contact with topical corticosteroids plays a pivotal role. Film-forming spray (FFS) can be a novel, alternate approach along with topical treatment in this area for carefully selected cases. This study aimed to evaluate the safety profile and physical characteristics of a novel formulation of an FFS in patients with psoriasis and eczema.

Methods

This open-label, multicentre, comparative study included subjects diagnosed with chronic plaque psoriasis requiring topical corticosteroid therapy or those with eczema necessitating its application and occlusion therapy. The study product was applied to two groups of subjects. For patients in group 1, the FFS was applied to the skin area affected by the dermatological condition, which was covered with ointment only. In the second group, the FFS was applied to the corresponding unaffected skin area. The FFS was applied for 60-90 seconds and was observed for two hours after the application. The subjects were evaluated for primary outcomes, including safety assessment, overall physical characteristics, and appearance of FFS from local skin effects. The secondary outcomes included physical appearance characteristics and overall patient satisfaction following the application of FFS at the target sites. Further statistical assessments were conducted using the SAS software version 9.4 (2023; SAS Institute Inc*., *Cary, North Carolina, United States).

Result

A total of 100 subjects were included in the study across 10 outpatient centers, of which 79% had psoriatic plaques and 21% had eczematous lesions. Primary outcomes showed a lack of appearance of clinical symptoms such as dryness, flakiness, or irritation. A total of 10% of subjects in group 1 had erythema, and 6% had a tingling sensation, which was transient and mild. The secondary outcomes showed that only 12% of subjects in Group 1 and 6% of subjects in Group 2 showed a feeling of stickiness at the application site. In group 1, 8% reported a cooling sensation, which disappeared in one and two minutes, and none experienced a cooling sensation in Group 2. The average drying time for FFS in subjects with dermatological conditions was 5.19 minutes compared to 1.51 minutes on unaffected skin. The film washability results indicated that 96% of subjects in group 1 reported complete removal in less than two minutes. At the end of the study period, the mean satisfaction score was 8.99. No significant adverse events were reported in the patients.

Conclusion

This study highlights the potential application of a novel formulation of FFS as a safe and well-tolerated option for enhancing uniform skin coverage with the topical corticosteroid in patients affected with psoriasis and eczema.

## Introduction

The impact of occlusion on skin barrier function and topical formulation is complex. When the skin barrier is impaired, subjects frequently experience skin effects such as dryness, itchiness, erythema, flakiness, cracks, and a roughened texture. Epidermal inflammation can further exacerbate the weakening of this protective barrier [1,2]. Topical corticosteroids for treating inflammatory and itchy skin conditions are commonly used for conditions like psoriasis, limited vitiligo, atopic dermatitis (AD), acute radiation dermatitis, lichen planus, and discoid lupus erythematosus [3]. Variations in transepidermal water loss (TEWL) reflect the current state of the skin barrier and also act as a subclinical biomarker for AD [4].

In India, psoriasis is prevalent among adults, ranging from 0.44% to 2.8%. Plaque psoriasis (PSO), the most prevalent form of psoriasis, accounts for over 80% of all psoriasis cases [5]. PSO located on exposed skin and sensitive areas such as the genitals, scalp, nails, palms, and soles can significantly impact patients' physical and emotional well-being [6]. There has been an increase in the prevalence of AD and eczema in India over the last four decades, with a current prevalence of 0.55% and 2.8%, respectively [7-9].

Topical corticosteroids are frequently used to reduce itching, improve skin appearance, and enhance the overall quality of life (QOL) [10]. The use of topical steroids is vital for managing chronic conditions, particularly in thickened, lichenified areas where sustained contact and enhanced penetration are crucial. Combining topical medication with occlusive therapies such as plastic wraps, bandages, gloves, and hydrocolloid dressings is common practice. It provides a protective barrier over the affected area, thereby reducing the risk of injury and external irritants. The combination may optimize the usage of topical formulation, and improve stratum corneum hydration [11,12]. Occlusive therapies can also limit the need for frequent reapplications, resulting in an overall improvement in patients' QOL.

Available topical dosage forms such as patches, ointments, creams, polymer foams, hydrogels, alginates, and hydrocolloids are commonly used. Occurrence of skin irritation is linked with some patches, primarily because of their occlusive nature. Patches are challenging to apply on curved surfaces, cause discomfort when peeled off, and may lack aesthetic appeal [13]. Semisolid preparations such as creams and ointments do not ensure persistent contact with the skin surface and can be easily wiped off by the patient’s clothing leading to repeated application. Moreover, these formulations can leave sticky and greasy residues after application, thereby reducing patient compliance [14]. Along with low visual appeal and cost, issues with occlusive therapies, such as hydrocolloid dressing, include irritation, folliculitis, excessive sweating, and koebnerization. Polymer films have several advantages in terms of film transparency, oxygen permeability, and adherence to the non-wounded skin [15]. Various dosage forms, including patches, gels, lotions, creams, ointments, and sprays, have been used to enhance the pharmacokinetic performance of topical drug administration [16].

Film-forming sprays (FFS) are formulations that transform into a film upon contact with the therapeutic site using polyacrylic co-polymers as a matrix [17]. Comprising inert substances, enhancers, and polymers in organic solvents, FFS comes in various types such as ordinary sprays, metered dose sprays, electrostatic sprays, and ultrasonic sprays. The choice of film-forming polymer is pivotal because it significantly affects the durability of the formulation, and these polymers must be able to create flexible, thin, transparent, and resilient films [18]. In the preparation of FFS, solid polymers do not directly enhance drug permeation; they form a transparent film that may aid drug permeation by providing occlusion. The film is removable with water and dissolves above pH 7 without causing skin irritation. Ethanol and isopropanol were used as solvents. When applied to the skin, the formulation evaporates rapidly, leaving a thin, transparent film in situ that adheres effectively to the area. FFS can be a novel, alternate approach along with topical treatment in this area for carefully selected cases. This innovative approach offers advantages, particularly in conditions like psoriasis, by ensuring more uniform and efficient skin coverage [16-18]. Based on preliminary data on safety profiles, which were previously conducted (data on file), spray-based solutions appear to acceptable safety profile on healthy human skin. Taking these data into consideration, the study was planned to evaluate the safety profile and physical characteristics of FFS in subjects with psoriasis.

## Materials and methods

Study design 

This prospective, open-label, comparative study was conducted on patients with plaque-type psoriasis and eczema diagnosed by treating clinicians in clinics/hospitals of Mumbai (Skin and Hair Clinic, Nerul; Sparsh Clinic, Nerul West; Rejoice Clinic, Dadar West; Skin Saga Centre for Dermatology, Andheri West; Dermatherapic Clinic, Khar West; and Dr Ghate's Skin & LASER Centre) and Guntur (Dr. Paruchuri Raja Ram Memorial Skin, Hair & Laser Centre), India. All participants provided their signed informed consent. The clinical trial was registered in Clinical Trials Registry-India (registration number: CTRI/2023/02/049491) and was approved by the Shah Lifeline Hospital And Heart Institute Ethics Committee (approval number: ECR/1588/Inst/MH/2021). It was conducted in accordance with the Good Clinical Practice standard and the Declaration of Helsinki.

Patient criteria

Eligible subjects of either gender aged between 18 to 65 years visiting outpatient clinics from November 2022 to March 2023 were included in the study. Subjects were diagnosed with mild to moderate plaque-type psoriasis (Psoriasis Area and Severity Index (PASI) score <10) for at least six months. The severity of psoriasis at the time of enrolment was assessed, and lesions located on the trunk, limbs, palms-soles, and flexures, which were localized, small (≤ 5cm), well-demarcated, and stable in morphology, were included at the baseline visit. The patients were undergoing treatment with topical corticosteroids continuously or intermittently for at least one week or more at the discretion of the treating dermatologists. 

Exclusion criteria included hypersensitivity to any component of the topical preparation, chronic or acute infection requiring treatment with systemic antibiotics, antivirals, antiprotozoals, or antifungal medications within four weeks prior to baseline, a history of no response to topical therapy, and ongoing use of moisturizer or skin softening ointment before enrolment. Guttate, erythrodermic, pustular psoriasis, or psoriatic arthritis were excluded from the assessment. Patients who were treated with cyclosporine or systemic corticosteroids before the study were excluded. Lesions on the scalp, face, intertriginous, genitalia, or other mechanically strained sites were excluded.

Study process

The study product is a proprietary formulation in the form of a sprayed solution containing a solid synthetic co-polymer (Eudragit S 100) as a matrix for film formation that forms a flexible and transparent film when it comes in contact with the target therapeutic site.

The patients provided informed consent and underwent screening for the study. Subjects were enrolled in the study at the enrolment visit to receive the test product. Screening and enrolment were considered as visit 1, which was either on the same day or on a different day. Subjects were assessed for coexisting conditions, vital signs, physical examination, concomitant medication, and side effects. The safety characteristics of the spray-based topical film-forming solution were further analyzed from a safety and tolerability perspective.

A total of 100 subjects were screened and further enrolled in the study. Body sides of eligible subjects were assigned to either group 1 or 2. Group 1 indicated individuals with affected areas that received steroid ointment followed by application of FFS. Group 2 indicated the corresponding opposite side of the unaffected area with the application of only FFS in the same individual.

In group 1, the treatment involved applying a thin layer of topical ointment 0.05% clobetasol propionate and 3% salicylic acid twice daily to the affected areas as per the instructions given by the dermatologist. The film-forming solution was sprayed by keeping its nozzle 3-5 inches away from the intended area to cover the area of previously applied medication completely. Repeated spraying was avoided. The solution could be set for a short time to form a flexible and transparent film in the area. 

In group 2, a comparable, unaffected, healthy area on the opposite side of the disease area was selected within the same subject. A similar process for applying the FFS was followed, and post-application local and overall effects were observed in patients for two hours.

Outcome measures

The primary outcomes included symptoms of local skin effects such as dryness, erythema, irritation, flakiness, and tingling sensation at the site of application in both groups, which were assessed using a visual analog scale (VAS) and a verbal rating scale (VRS) by the investigator.

Secondary outcomes included physical characteristics such as cooling sensation, the time required for drying, the time needed to wash the film (washability of the film), a feeling of stickiness (gluey) in appearance, and overall patient satisfaction. The overall satisfaction score was obtained using a VAS, which was reported by patients and further evaluated by the investigator. Prespecified safety outcomes included treatment-emergent adverse events (TEAEs), serious adverse events (SAEs), vital signs, and physical examinations. Outcomes were assessed at baseline and the end of the treatment.

Water washability was evaluated in the dried film. The film was washed with running water, and the time required to clean the movie was assessed. The drying time of the film determines how rapidly it forms once the solution is sprayed. To determine whether the film had dried, a glass plate was placed against it without being touched. The film was considered to have dried if there was no sign of water adherence to the glass.

Statistical analysis

All participants were assessed for the primary and secondary outcomes in statistical analysis. Baseline characteristics were summarized as means and standard deviations for continuous variables and as counts and percentages for categorical variables. All analyses were conducted using the SAS software version 9.4 (SAS Institute Inc., Cary, North Carolina, United States).

## Results

Demography and baseline data

A total of 100 subjects were enrolled during the study period at 10 centers of outpatient clinics in India, of which 79 patients had psoriasis and 21 had eczema (AD) (Table 1). Of the included patients, 46% were male and 54% were female, with a mean age of 40.31 (±12.31) years. Patients presented with a mean duration of disease of 15.34 (±14.22) years. All subjects completed the study without discontinuing the product application and evaluations. On the day of enrolment, the FFS was sprayed on affected areas after the application of ointment in each subject. Most lesions were located on extensor surfaces of the knees and elbows (26/40; 65%), where total number of sites of knee and elbow (23+17) is 40, and the plantar aspect of the foot (12/16; 75%), where total number of lesions on feet is 16, whereas legs were involved in 14% (n=14) of cases and other body parts like thighs, upper arm, shoulder, and back were involved in 13% (n=13) of cases (Table 2). The physical characteristics and appearance of the FFS were observed for two hours following its application.

**Table 1:** Baseline characteristics of study participants

Characteristics	Mean (SD)	Min	Max
Age (years)	40.31 (12.31)	19	65
Gender (male/female)	46/54	-	-
Weight (kg)	68.29 (13.66)	40.6	110
Height (cm)	160.75 (8.32)	125	179.8
Body Mass Index (kg/m^2^)	26.44 (4.57)	17.95	42.44

**Table 2:** Summary of areas selected for film-forming spray application Data presented as number (n)

Selected areas	Total (N=100), n	Psoriasis (N=79), n	Eczema (N=21), n
Knee	23	18	5
Shoulder	8	7	1
Elbow	17	13	4
Palm	5	5	0
Foot	16	9	7
Leg	14	14	0
Thigh	2	2	0
Hand	12	12	0
Back	2	2	0
Upper arm	1	1	0

Clinical parameters

The clinical parameters were observed for two hours after the application of FFS. Dryness, erythema, irritation, flakiness, and tingling sensation were assessed as primary outcomes using the VAS and VRS by the investigator (Table 3). The results showed no signs of dryness, flakiness, or irritation in any of the subjects, while 10 (10%) subjects in group 1 had erythema and six (6%) subjects had a tingling sensation. Group 2 did not report any post-application symptoms. The erythema and tingling sensations experienced were transient and mild. None of the subjects required additional treatment to reverse their symptoms throughout the study.

**Table 3:** Summary of primary outcomes Data presented as number (n)

Outcomes		Group 1, n	Group 2, n
Erythema		10	0
Severity		Mild	0
Time required for the symptom to disappear	1 minute	5	0
	2 minutes	5	0
Tingling sensation		6	0
Severity		Mild	0
Time required for the symptom to disappear	1 minute	3	0
	2 minute	3	0

The feeling of stickiness (gluey) in appearance, sensation of cooling, time required for drying, and washability of the film were evaluated as secondary endpoints (Table 4). Only 12% (n=12) of subjects in group 1 and 6% (n=6) of subjects in group 2 showed a feeling of stickiness (gluey). In group 1, 8% (n=8) of the subjects reported a cooling sensation, which disappeared in one and two minutes. None of the subjects in group 2 experienced a cooling sensation.

**Table 4:** Summary of secondary outcomes Data presented as number (n)

Outcomes		Group 1, n	Group 2, n
Feeling of stickiness (gluey) in appearance		12	6
Clinical parameter resolved/disappeared		12	6
Time required for the symptom to disappear	1 minute	8	4
	2 minute	4	2
Cooling sensation		8	0
Clinical parameter resolved/ disappeared		8	0
Time required for the symptom to disappear	1 minute	5	0
	2 minute	3	0

**Figure 1:**
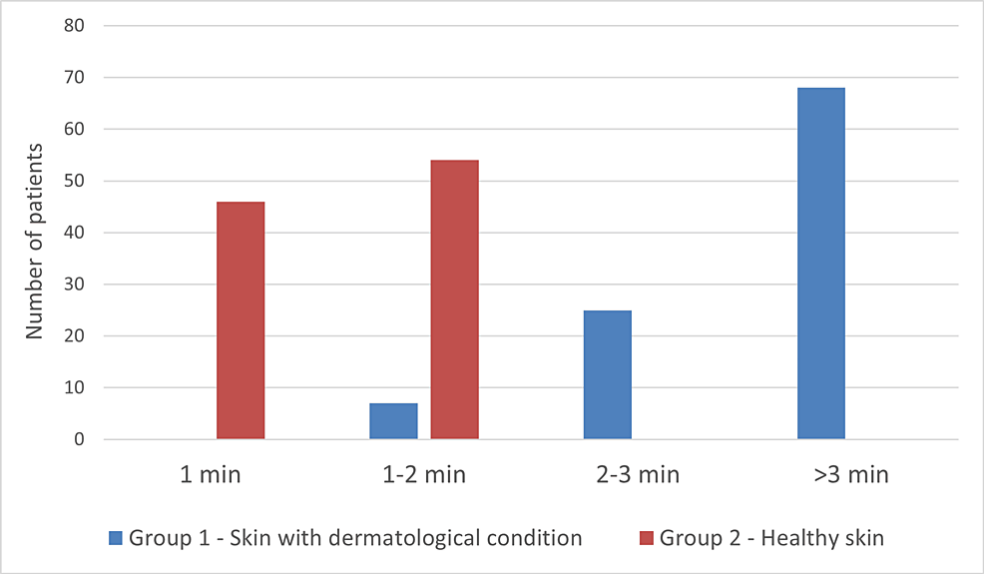
Time required to dry the film Standard deviation (SD) in skin with dermatological condition (group 1) = 1.85; SD in healthy skin (group 2) = 0.48 There was a statistically significant difference between Group 1 and Group 2 (p < 0.0001).

**Figure 2:**
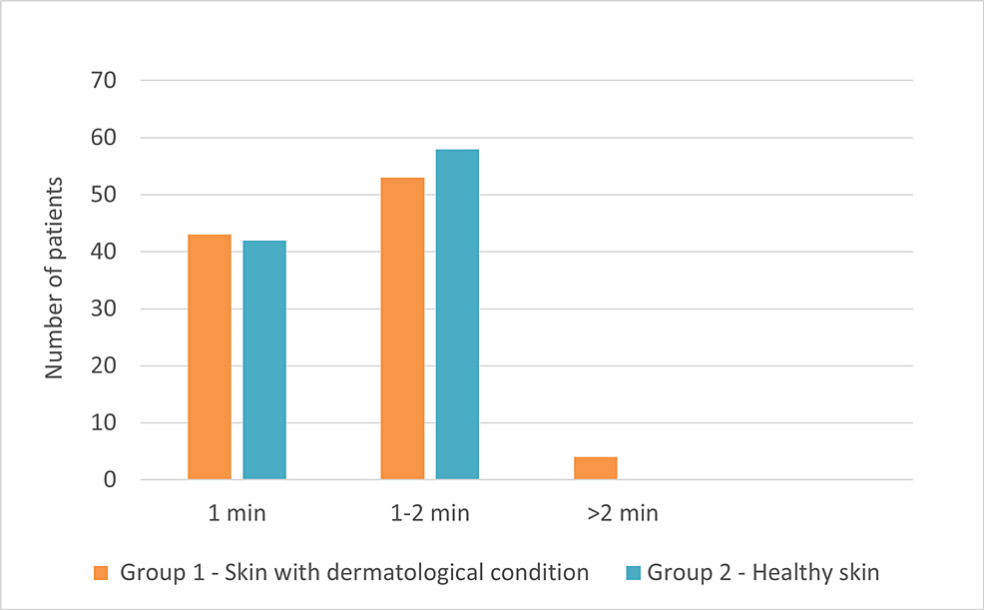
Time required to wash the film There was no significant difference in the time required to remove the film (less than two minutes) from the target site between group 1 and group 2 (p = 0.693).

In groups 1 and 2, the minimum time required to dry FFS was two minutes and one minute, respectively. The maximum time needed to dry FFS was eight minutes in group 1 and two minutes in group 2 (Figure 1). As a result, the average drying time for FFS on skin with dermatological conditions was 5.19 minutes and 1.51 minutes for unaffected skin (p<0.0001). The washability of the film results revealed that 96% (n=96) of subjects in group 1 showed that the film was entirely removed in less than two minutes. Only 4% (n=4) of subjects took longer than two minutes to wash off the film entirely (Figure 2). In group 2, all participating subjects indicated that the film was washed off within two minutes.

Subgroup analysis

Subgroup analysis of dermatological conditions highlighted the differences among age groups. When examining the drying time within specific age groups, we observed that nine subjects each in the age groups of 18-30 and 31-40 years, had the highest proportion of subjects reporting a drying time of three to four minutes and seven to eight minutes, respectively, while 32% (n=32) of subjects reported a drying time of five to six minutes. The erythema analysis revealed that subjects in the age group of 31-40 years had the highest number of reported cases (five subjects), followed by those in the age group of 41-50 years (three subjects). The cooling sensation was most frequently reported by subjects aged 51-60 years (six subjects). The age group of 18-30 years had the highest number of subjects reporting tingling sensations (three subjects),

The overall satisfaction rate was analyzed at the end of the study. The highest satisfaction score of 10 was reported by 34 (34%) subjects, while five (5%) subjects reported a minimum VAS score of 7. The mean satisfaction score analyzed was 8.99. 

Vital signs were evaluated, including blood pressure, pulse, respiration rate, and temperature. All the results were within the clinically acceptable ranges. Physical examination was normal for each subject during each visit. No adverse events or SAEs were reported during the trial.

## Discussion

This comparative study evaluated the safety and tolerability of FFS in patients with psoriasis and eczema. The results showed that FFS provided a uniform layer over the ointment applied area in patients with dermatological conditions.

Traditional topical formulations and occlusive dressings are frequently used to treat various dermatological conditions and efficiently deliver pharmaceuticals to the skin surface [19]. Occlusive dressings such as plastic wrap, bandages, or gloves trap heat and moisture, aid in wound healing, and enhance the penetration of topical drugs through the skin [20]. Notably, in the topical treatment of psoriasis, patients should receive regular motivation and reinforcement regarding the correct application and frequency. It is essential to ensure consistent adherence over time and to achieve optimal results. Additionally, it is necessary to understand and address patient expectations individually. Compared to other topical dosages, sprays offer several advantages such as practical use, low incidence of irritation, sterility of the dosage, excellent coverage of the skin or affected part, even distribution of the drug when applied, and adjustable dosage. To overcome these limitations, the development of FFS has emerged as a valuable solution for dermatological treatment. When applied to the skin, FFS forms a protective and nearly invisible layer that acts as a barrier between medication and external factors, ensuring that it remains in contact with the skin for an extended duration [21].

Our study included 100 subjects with mild and moderate PSO and eczema requiring topical corticosteroid ointment application and occlusion therapy. The study results demonstrated that FFS did not induce dryness, flakiness, or irritation in the subjects and did not cause any significant adverse effects on the skin. While some subjects in group 1 experienced erythema and tingling sensations, the symptoms were mild and resolved spontaneously without the need for intervention. This observation highlights the self-limiting nature of reported erythema and tingling sensations, emphasizing the overall safety and tolerability of FFS. A comparison of physical parameters showed that a relatively lower percentage of subjects in both group 1 and group 2 reported a feeling of stickiness (gluey). These symptoms were transient as they disappeared within a short timeframe. The FFS did not induce persistent cooling sensations. None of the subjects in group 2 (healthy skin) experienced a cooling sensation, suggesting that this effect might be associated with dermatological conditions only. The results indicated that the film was comparably washable in both subject groups and comprehensively understood the product's characteristics and suitability for dermatological conditions.

The subgroup analysis for dermatological conditions revealed notable differences in drying time among different age groups. Younger subjects experienced a slightly longer drying time whereas FFS dried more quickly in older subjects; the sensation of feeling sticky varied across the age groups. The subjects in the age group of 31-40 years experienced erythema, while the older subjects were more likely to perceive a cooling sensation after using the product. The subjects in the age groups 18-30 years were more sensitive to tingling sensations.

Most of the subjects experienced excellent patient satisfaction with the intervention. The overall satisfaction mean score was 8.99, suggesting the safety acceptability of the FFS being studied. Regarding tolerability, FFS did not lead to any adverse drug reactions; minor side effects such as erythema were resolved without further management, assuring evidence of the safety and user-friendliness of FFS. FFS offers a quick drying time with consistent washing-off properties, making it a promising option for applications in various dermatological conditions.

A study by Jacob et al., which included 30 subjects affected with psoriasis from the southern region of India, sought to assess the effectiveness of cling film dressing to traditional non-occlusive topical therapy in reducing the severity of psoriatic illness [20]. The results revealed a statistically significant decrease in disease severity among the cling film-treated group: 9.88 (p<0.05) and 4.02 (p<0.001), respectively. Furthermore, a clinical and immunohistology study on occlusive therapy assessed the clinical efficacy of prolonged occlusion dressing, fluocinonide ointment, or a combination of the two [11]. The results showed that the combination of fluocinonide ointment and occlusion produced more significant improvement than either treatment alone (p<0.001).

This is the first FFS study conducted in the Indian setting, with an emphasis on the assessment of its safety parameters in subjects with dermatological conditions. This study had a few limitations such as self-reported outcomes and in terms of providing comparative data with other existing formulations, as this is a novel product for enhancing skin coverage in affected areas. Limitations of the study also include the lack of direct comparison with an ointment /cream without FFS to support the overall benefit which is being planned in the next phase of the study. Additional research is needed to evaluate the effectiveness of this novel formulation using a larger sample size with a range of additional pertinent dermatological conditions. These results encourage further research to establish a more extensive database of various dermatological conditions.

## Conclusions

The study highlights the potential use of the polyacrylic co-polymer-based FFS as a safe and tolerable approach in a subset of patients with psoriasis and eczema for uniformly enhancing skin coverage of the topically applied creams and ointments. A spray-based solution was well tolerated and had less drying time, strongly supporting its clinical adoption in managing certain conditions in patients affected with psoriasis. The high acceptance rate among the patients highlights its convenience to use, emphasizing its potential as a valuable addition to clinical dermatology. The promising outcomes warrant further exploration and clinical application, potentially offering relief and improved quality of life to subjects managing challenging skin conditions.
